# Pretreatment with 2-(4-Methoxyphenyl)ethyl-2-acetamido-2-deoxy-β-D-pyranoside Attenuates Cerebral Ischemia/Reperfusion-Induced Injury *In Vitro* and *In Vivo*

**DOI:** 10.1371/journal.pone.0100126

**Published:** 2014-07-03

**Authors:** Xia Chen, Aiqing Deng, Tianqiu Zhou, Fei Ding

**Affiliations:** 1 Basic Medical Research Centre, Medical School, Nantong University, Nantong, China; 2 Department of Pharmacy, Affiliated Hospital of Nantong University, Nantong, China; 3 Department of ophtalmology, Affiliated Hospital of Nantong University, Nantong, China; 4 Jiangsu Key Laboratory of Neuroregeneration, Nantong University, Nantong, China; National University of Singapore, Singapore

## Abstract

Salidroside, extracted from the root of *Rhodiola rosea* L, is known for its pharmacological properties, in particular its neuroprotective effects. 2-(4-Methoxyphenyl) ethyl-2-acetamido-2-deoxy-β-D- pyranoside (GlcNAc-Sal), an analog of salidroside, was recently synthesized and shown to possess neuroprotective properties. The purpose of the current study was to investigate the neuroprotective effects of GlcNAc-Sal against oxygen–glucose deprivation-reperfusion (OGD-R)-induced neurotoxicity in vitro and global cerebral ischemia-reperfusion (GCI-R) injury in vivo. Cell viability tests and Hoechst 33342 staining confirmed that GlcNAc-Sal pretreatment markedly attenuated OGD-R induced apoptotic cell death in immortalized mouse hippocampal HT22 cells. Western blot, immunofluorescence and PCR analyses revealed that GlcNAc-Sal pretreatment restored the balance of pro- and anti-apoptotic proteins and inhibited the activation of caspase-3 and PARP induced by OGD-R treatment. Further analyses showed that GlcNAc-Sal pretreatment antagonized reactive oxygen species (ROS) generation, iNOS-derived NO production and NO-related apoptotic cell death during OGD-R stimulation. GCI-R was induced by bilateral common carotid artery occlusion (BCCAO) and reperfusion in mice in vivo. Western blot analysis showed that GlcNAc-Sal pretreatment decreased the expression of caspase-3 and increased the expression of Bcl-2 (B-cell lymphoma 2)/Bax (Bcl-2-associated X protein) induced by GCI-R treatment. Our findings suggest that GlcNAc-Sal pretreatment prevents brain ischemia reperfusion injury by the direct or indirect suppression of cell apoptosis and GlcNAc-Sal could be developed as a broad-spectrum agent for the prevention and/or treatment of cerebral ischemic injury.

## Introduction

Cerebral ischemic injury is one of the leading causes of death and disability. Ischemic stroke, which results in insufficient supply of glucose and oxygen to brain tissues, causes significant damage to cells associated with oxidative stress, the regulation of pro-apoptotic and anti-apoptotic factors, and dysfunction of neuronal signaling pathways [1], [2], [3], [4]. The rapid initiation of reperfusion therapy is an effective strategy to reduce the infarct area and minimize the behavioral deficits resulting from ischemia. However, reperfusion itself is associated with injury as a result of the overproduction of reactive oxygen species and overloading of calcium that occur in the early reperfusion period [5], [6], [7]. The oxygen-glucose deprivation followed by reperfusion (OGD-R) model mimics the key pathophysiological events of ischemia in vitro and enables the dissection of cellular events without affecting oxygen and metabolites [8]. Moreover, it provides a method to test the neuroprotective effects of pharmacological compounds [9], [10]. Global cerebral ischemia-reperfusion (GCI-R), which is widely used to evaluate the relationship of chronic cerebral hypoperfusion with cognitive ability [11], [12], has helped understanding of the role of cerebral hypoperfusion in neurodegenerative diseases [13]. The hippocampus is responsible for many central nervous system functions including cognition, learning, and memory, but it is also one of the most vulnerable brain regions as regards to various neurological insults such as hypoxia–ischemia, seizure and prolonged stress [14]. Based on these considerations, hippocampus is widely used to explore the neuroprotective effects of pharmacological compounds to brain ischemic induced by OGD-R in vitro or GCI-R in vivo.

Even though many different compounds have been proven to reduce the size of brain infarct in animal studies, replication of the experiments have regularly failed in humans. Either the toxic side effects, which have overridden the neuroprotective potential of the compounds determined in animals, or a limited time window for human therapy may explain the unsuccessful clinical trials. Many scholars were interest in searching natural origin drug with no or tolerable side effects which can treat cerebral ischemia-reperfusion injury.

**Figure 1:**
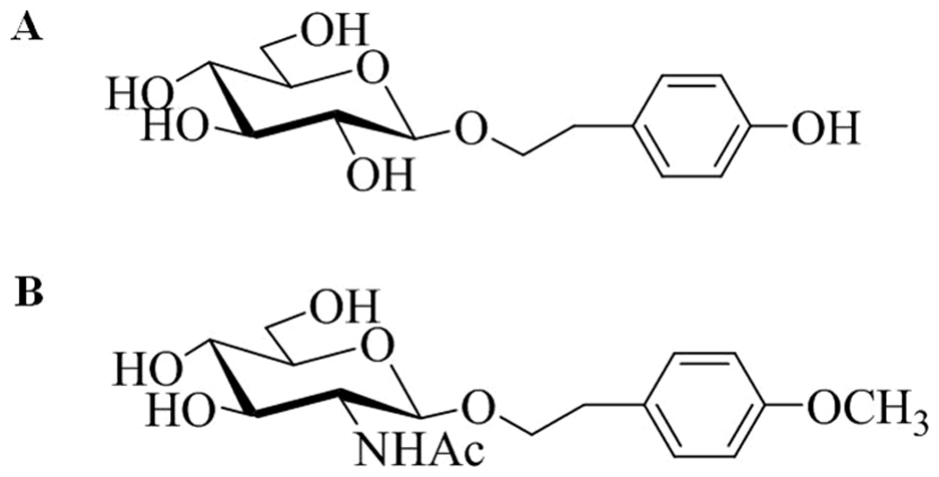
Chemical structure of salidroside and GlcNAc-Sal. (A) p-hydroxyphenethyl-β-D- glucoside. (B) 2-(4-Methoxyphenyl)ethyl-2-acetamido-2-deoxy-β-D-pyranoside.

Salidroside (Fig. 1A) is an active compound extracted from the root of *Rhodiola rosea* L that has been used in traditional Tibetan medicine as an adaptogen. This compound is known to possess pharmacological properties including anti-oxidative, neuroprotective and anti-depressive effects [15], [16], [17], [18], [19], [20], [21], [22], [23]. However, the sources of wild *Rhodiola rosea* L are on the edge of exhaustion. Therefore, considerable effort has been devoted to the synthesis and structure modification of salidroside. Our group synthesized a salidroside analog 2-(4-Methoxyphenyl)ethyl-2-acetamido-2-deoxy-β-D-pyranoside (GlcNAc-Sal) (Fig. 1B) and showed that it has pharmacological properties including anti-oxidation and anti-apoptosis, and its protective effects was shown to be superior to that of salidroside [24], [25], [26].

In order to provide a new window into the pharmacological properties of GlcNAc-Sal, the present study was designed to investigate neuroprotective effects of GlcNAc-Sal on OGD-R-induced HT22 cell death in vitro and GCI-R-induced hippocampal damage in vivo and further explored the underlying mechanisms. We hope to expand the understanding of the potential therapeutic value of salidroside for cerebral ischemia injury.

## Materials and Methods

### Cell culture and treatment

Immortalized mouse hippocampal HT22 cells (a subclone of HT4, originating from mouse hippocampus), a generous gift from Department of Pharmacology and Laboratory of Aging and Nervous Diseases, Soochow University School of Pharmaceutical Science [27], were plated and maintained in high-glucose Dulbecco's Modified Eagle's Medium (DMEM, Gibco, Grand Island, NY) supplemented with 10% fetal bovine serum (FBS, Sijiqing, Hangzhou, China), 100 U/mL penicillin, and 100 U/mL streptomycin in a humidified atmosphere of 5% CO_2_ and 95% air at 37°C.

To induce OGD-R injury, cells were rinsed twice and incubated in a glucose-free Hank's balanced salt solution (HBSS) composed of 140 mM NaCl, 1.2 mM MgSO_4_, 1.7 mM CaCl_2_, 3.5 mM KCl, 10 mM HEPES, 5 mM NaHCO_3_, 0.4 mM KH_2_PO_4_ (pH 7.3). Then, the cultures were placed into a specialized, humidified chamber filled with 95% N_2_ and 5% CO_2_ at 37°C for the indicated times. Controls were incubated with the HBSS buffer containing 5.6 mM glucose in a humidified incubator with 5% CO_2_ at 37°C for the same times as the OGD-R cultures. After the challenge, cultures were transferred to normal culture medium and returned back to normoxic conditions for 24 h to induce reperfusion. The indicated concentrations of GlcNAc-Sal was added to the culture medium 24 h before OGD-R and maintained until the end of recovery.

SNP (sodium nitroprusside, a NO donor) injury was induced by pretreating HT22 cells with GlcNAc-Sal at different concentrations for 24 h followed by treatment with SNP for an additional 12 h. After the cell treatments, cell viability and Hoechst 33342 staining assays were performed.

### Cell viability test

Cell viability was assessed using the MTT (Sigma, St. Louis, MO) assay as described previously. Briefly, HT22 cells were seeded in 96-well culture plates at a density of 1×10^4^ cells/well. After pretreatment with GlcNAc-Sal for 24 h and exposure to OGD-R or SNP, 100 µL of MTT solution was added to each well and incubated at 37°C for 4 h followed by 100 µL of 20% sodium dodecyl sulfide (SDS) solution to dissolve the precipitate for 20 h. Absorbance was measured by spectrophotometry at 570 nm with an ElX-800 Microelisa reader (Bio-Tek Inc., Winooski, VT). The data were expressed as a percentage of the controls.

Cytotoxicity was quantified by measuring the lactate dehydrogenase (LDH) released to the medium using a LDH-Cytotoxic test kit (Genmed, Westbury, NY) according to the manufacturer's instructions. Briefly, HT22 cells were seeded in 96-well culture plates at a density of 1×10^4^ cells/well. Then, control cells were treated with 10 µL of cell lysis solution and designated as untreated cells, whereas an equal volume of balanced solution was added to treated cells. After 1 h of treatment, 50 µL of the supernatant was transferred to the corresponding well of a fresh 96-well plate and mixed with 50 µL of the LDH substrate. After incubation for 0.5 h at room temperature, the reaction was stopped by addition of 10 µL of stop buffer and the absorbance was measured at 490 nm with an ElX-800 Microelisa reader (Bio-Tek, Inc.). The percentage of LDH release was calculated using the following formula: (absorbance of sample ÷ absorbance of maximum enzyme activity) ×100.

Cells of different groups were treated as described above. And the morphology of cells was monitored under an inverted phase-contrast microscope (Olympus, Tokyo, Japan) and photographed.

### Hoechst 33342 staining

HT22 cells were cultured on 24-well culture plates at a density of 1×10^5^ cells/cm^2^. Cells were treated as described above and fixed in 4% paraformaldehyde in PBS at room temperature for 20 min. After staining with 10 µg/mL Hoechst 33342 (Sigma, St. Louis, MO) for 10 min, the cells were observed under a DMR fluorescence microscope (Leica Microsystems, Wetzlar, Germany) with excitation at 340 nm and emission at 510 nm. HT22 cells showing fragmented or condensed DNA were counted as apoptotic cells, and the ratio of apoptotic cells to total cells was calculated.

### Immunofluorescence analysis of the expression of Bcl-2 and Bax

Cells were fixed with 4% paraformaldehyde at room temperature for 30 min and washed twice with PBS. After permeabilization with 0.5% Triton X-100 and blocking with 1% bovine serum albumin in PBS for 1 h, the cells were incubated with mouse anti-Bcl-2 and anti-Bax monoclonal antibodies (1∶100, Santa Cruz Biotechnology Inc., CA, USA) overnight at 4°C, followed by incubation with Cy3-labeled goat anti-mouse IgG (H+L) (Beyotime Institution of Biotechnology, Haimen, China) for 2 h at 37°C. The cells were then incubated with Hoechst 33342 for 10 min and examined under a fluorescence microscope (488 nm filter; OLYMPUS BX51, Japan).

### Reverse transcription-PCR

Total RNA was isolated using RNAsimple Total RNA Kits (TIANGEN, Beijing, China) and subjected to reverse-transcription to synthesize cDNA using the RevertAid First Strand cDNA Synthesis Kit (Fermentus Life Science, Ontario, Canada). The primer sequences for each gene are listed in Table 1 [22], [28]. The PCR conditions were 94°C for 30 s (denaturation), 55°C (Bcl-2, Caspase-3 and GAPDH), and 52°C (Bax) for 45 s (annealing) and 72°C for 1 min (extension) for a total of 35 cycles (Bcl-2, caspase-3 and GAPDH) and 30 cycles (Bax). The PCR products were separated by 1.5% agarose gel electrophoresis and visualized by ethidium bromide staining. Images were acquired and quantified using Image J software.

**Table 1:** Summary of the reverse transcription-PCR primers.

Gene	Forward primer (5’-3’)	Reverse primer (5’-3’)	Product
Bcl-2	TGGGATGCCTTTGTGGAACTA	GCTGATTTGACCATTTGCCTG	328bp
Bax	AACATGGAGCTGCAGAGGAT	TCCCGAAGTAGGAAAGGAGG	283bp
Caspase3	GGACCTGTGGACCTGAAAAA	CGGGATCTGTTTCTTTGCAT	396bp
GAPDH	CAAGGTCATCCATGACAACTTTG	GTCCACCACCCTGTTGCTGTAG	496bp

### Measurement of intracellular reactive oxygen species

Intracellular reactive oxygen species (ROS) formation was measured using the non-fluorescent probe 2′,7′-dichlorofluorescein diacetate (DCFH-DA, Beyotime Institution of Biotechnology, Haimen, China). DCFH-DA diffuses into cells where it is deacetylated by esterases to form the non-fluorescent compound DCFH. DCFH reacts with ROS to form the fluorescent product DCF, which is trapped inside the cells. Briefly, HT22 cultured in plates were loaded with 10 µM DCFH-DA in serum-free medium at 37°C for 30 min. After DCFH-DA treatment, the chemicals were removed and the plates were washed three times with serum-free medium. The fluorescence generated by ROS was visualized with a fluorescence microscope (488 nm filter, OLYMPUS BX51, Japan). The fluorescence intensity of eight fields per dish was measured and ROS level was quantified using Image-pro Plus software. Three parallel experiments were performed and the results were expressed as mean values [29].

### Nitrite assays

Levels of NO was determined by measuring the accumulated level of nitrite (an indicator of NO) in the supernatant using a colorimetric reaction with Griess reagent. A nitrite detection kit (Beyotime Institution of Biotechnology, Haimen, China) was used according to instructions provided by the manufacturer. Briefly, 50 µL of culture medium were mixed with an equal volume of Griess reagents I and II at room temperature and NO concentration was determined by measuring the absorbance at 540 nm on a microplate reader. Nitrite concentration was calculated with reference to a standard curve of sodium nitrite (0–100 µM) [30].

### Nissl staining

Anesthetized animals (six mice from each group) were perfused with normal saline followed by 4% paraformaldehyde. Brains were removed, post-fixed for 24 h in the same fixative solution at 4°C, dehydrated and then embedded in paraffin blocks. Coronal sections (15 µm) of the brain were obtained and stained with 0.5% cresyl violet (Sigma, St. Louis, MO), dehydrated through a graded alcohol series (70%, 80%, 90%, and 100%×2), placed in xylene, and covered with a coverslip after the addition of Histomount media. The neurons in the hippocampal CA1 region were observed with a light microscope (Olympus BX51, Japan).

### Surgical procedure and drug administration

Male Kunming mice (6–8 weeks old, 25–30 g), obtained from the Experimental Animal Center of Nantong University, were used in this study. The experimental procedures were conducted in accordance with institutional animal care guidelines and approved ethically by the Administration Committee of Experimental Animals, Jiangsu Province, China. Efforts were made to minimize animal suffering and to reduce the number of subjects used.

The mice were assigned to a sham group or a bilateral common carotid artery occlusion (BCCAO) surgery group to induce GCI-R as previously described [31], [32], generating three treatment groups (each group n = 12): Sham (sham + saline), GCI-R (GCI-R + saline), and G + G (GCI-R + GlcNAc-Sal). Briefly, mice were anesthetized with chloral hydrate (350 mg/kg, i.p.). After a median incision was made in the neck skin, both common carotid arteries were exposed and occluded with cotton thread for 60 min, after which the thread was removed to allow reperfusion for 4 h. The animals were then killed for the next assays. Mice receiving the same operation without arterial occlusion served as sham-operated controls.

GlcNAc-Sal (800 µM, 2 µL) or vehicle (saline, 2 µL) was injected into the right lateral ventricle at 1 µL/min [stereotaxic coordinates: 0.2 mm caudal to the bregma and 1.0 mm lateral to midline at a depth of 4 mm from the skull surface at the bregma, based on the mouse atlas] under anesthesia [chloral hydrate (350 mg/kg, i.p.)] 30 min before transient global cerebral ischemia.

### Western blot analysis

#### Expression of Bcl-2, Bax, caspase-3, PARP and iNOS after exposed to OGD-R in HT22 cells

After exposure to different treatments, cells were collected and lysed in ice-cold buffer (tris-(hydroxymethyl) aminomethane 50 mmol/l, pH 7.4, NaCl 150 mmol/l, 0.5% Triton X-100, edetic acid 1 mmol/l, phenylmethylsulfony fluoride 1 mol/l, and aprotinin 5 mg/l), and then centrifuged at 12,000 g for 5 min at 4°C. The supernatant was collected and the protein concentration was determined using the BCA-100 Protein Quantitative Analysis Kit (Beyotime, Haimen, China). Equal amounts of proteins were separated by 12% SDS-PAGE and transferred to nitrocellulose membranes (Millipore, Bedford, MA, USA) followed by blocking in 5% non-fat milk for 1 h. The membranes were then incubated overnight at 4°C with rabbit polyclonal anti-Bcl-2, rabbit polyclonal anti-Bax, rabbit monoclonal anti-PARP, rabbit monoclonal anti-caspase-3 or rabbit polyclonal anti-iNOS antibodies (1∶1000, Cell Signaling Technology, Beverly, MA). After washing with TBS/T (TBS with 0.05% Tween 20), membranes were incubated in IRDye 680-conjugated affinity purified goat anti-rabbit IgG (1∶5000, Bioworld Technology, Inc., Minneapolis, MN, USA)) at room temperature for 1 h. The images were scanned with the Odyssey infrared imaging system (LI-COR) and quantified with Multi Gauge. GAPDH (Anti-GAPDH antibody, Bioworld, Minneapolis, MN, USA), was used as an internal reference.

#### Expression of Bcl-2, Bax and caspase-3 after exposed to GCI-R in hippocampus of mice

After different treatments, mice were sacrificed. The entire brains were removed quickly for dissecting the hippocampus. The hippocampal tissue was homogenized in an ice-cold buffer, centrifuged at 15, 000 rpm at 4°C for 10 min. The supernatant was then collected as total protein and subjected to western blot analysis the expression of Bcl-2, Bax and caspase-3 as described previously.

### Statistical analysis

Data were expressed as mean ± SEM. Statistical significance was determined by one-way analysis of variance (ANOVA) and subsequent Tukey's test. Differences were considered significant at *p*<0.05.

## Results

### Protective effects of GlcNAc-Sal pretreatment on OGD-R-induced apoptosis in HT22 cells

**Figure 2:**
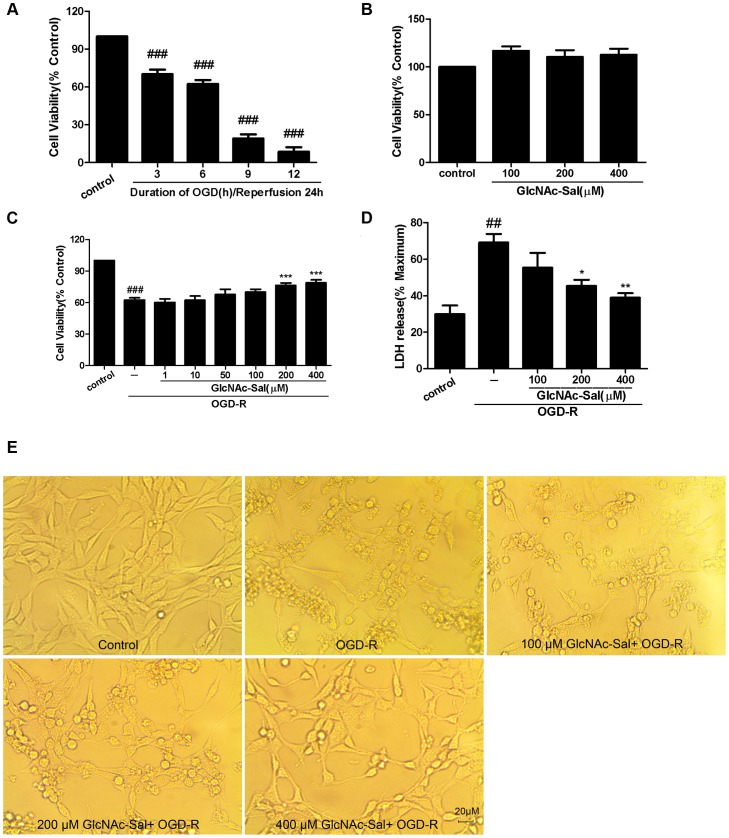
Protective effects of GlcNAc-Sal on OGD-R-induced cytotoxicity in HT22 cells. Cell viability was determined by the MTT assay and LDH release assay. (A) HT22 cells were exposed to OGD insults of different durations followed by 24 h of reperfusion. (B) HT22 cells were exposed to different concentrations of GlcNAc-Sal for 24 h. The effect of GlcNAc-Sal on the viability of HT22 cells exposed to OGD-R stimulation was analyzed by the MTT assay (C) and LDH release assay (D). (E) Morphological alterations in HT22 cells. All data were expressed as mean ±SEM of three independent experiments performed in triplicate. ^##^ *p*<0.01, ^###^ *p*<0.001 vs. control; *p<0.05, **p<0.01 vs. OGD-R alone.

The viability of HT22 cells exposed to OGD insult was assessed using the MTT assay, which showed that the cell death caused by OGD occurred in a time-dependent manner (Fig. 2A). Exposure to OGD for 6, 9, 12 h followed by 24 h of reperfusion resulted in about 40%, 80% and 90% loss of cell viability. Pretreatment with GlcNAc-Sal could attenuate the insult by OGD for 6 h followed by 24 h of reperfusion but could not reverse the serious insult by OGD for 9 h and 12 h followed by 24 h of reperfusion (data not shown). Based on the above considerations, we used OGD treatment for 6 h followed by 24 h of reperfusion to induce cell damage in the present study.

GlcNAc-Sal pretreatment alone did not affect cell viability (Fig. 2B). OGD-R insult decreased the viability of HT22 neurons by 65.10±3.59%, and treatment with GlcNAc-Sal at low concentrations (e.g. 100 µM) had no neuroprotective effect (Fig. 2C). GlcNAc-Sal at 200 and 400 µM, however, significantly prevented OGD-R induced damage in cultured HT22 cells, and restored cell viability to 76.34±2.29% and 78.81±2.99%, respectively, showing dose-dependent protective effects (Fig. 2C).

OGD-R insult significantly increased the release of LDH from 29.87±4.76% in the control group to 69.09±4.76%. Furthermore, decreases in LDH release from 69.09±4.76% to 55.45±7.96%, 45.33±3.36% and 38.91±2.48% in response to GlcNAc-Sal treatment at 100, 200 and 400 µM, respectively, confirmed its protective effect (Fig. 2D).

Light micrographs confirmed the neuroprotective effects of GlcNAc-Sal (Fig. 2E). Untreated HT22 cells (control) showed an intact cell membrane and good refraction. HT22 cells exposed to OGD-R showed significant damage, as evidenced by the disappearance of cellular processes and decreased refraction. GlcNAc-Sal pretreatment significantly inhibited OGD-R induced cell damage in HT22 cells, as observed in the micrographic images.

**Figure 3:**
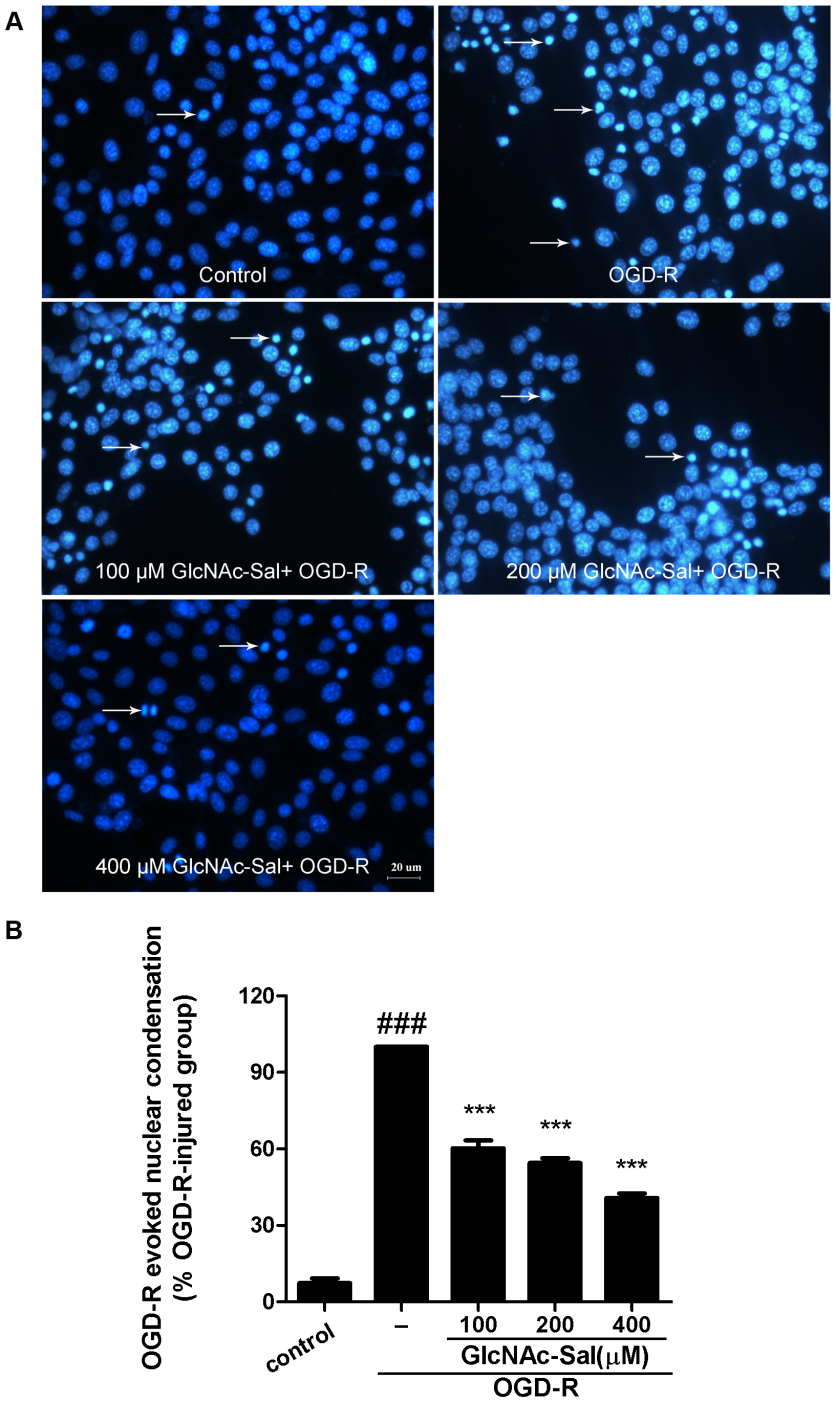
GlcNAc-Sal protects HT22 cells against OGD-R-induced apoptosis. (A) Hoechst 33342 staining was used to detect the morphological features of apoptosis in HT22 cells (condensed chromatin and fragmented nuclei). Arrowheads indicted apoptosis cells. (B) The rate of nuclear condensation in response to OGD-R stimulation was calculated (^###^ *p*<0.001, compared with untreated control cells, *** *p*<0.001 compared with OGD-R-treated cells). All data were expressed as mean ± SEM of three independent experiments.

Because the MTT and LDH release assays failed to distinguish between necrosis and apoptosis, we performed morphological examinations to determine the underlying mechanism of OGD-R induced cell death. Hoechst 33342 staining (Fig. 3A and B) showed that in response to the OGD-R insult, approximately 35% of HT22 cells displayed typical morphological features of apoptosis including chromatin condensation, nuclear shrinkage, and the formation of a few apoptotic bodies. Pretreatment with 100, 200, and 400 µM GlcNAc-Sal reduced the percentage of apoptotic cells by approximately 38%, 45% and 60%, respectively.

Taken together, these results suggest that GlcNAc-Sal pretreatment attenuates OGD-R induced apoptotic cell death in cultured HT22 cells.

### GlcNAc-Sal pretreatment affects OGD-R induced expression of apoptosis genes and proteins in HT22 cells

**Figure 4:**
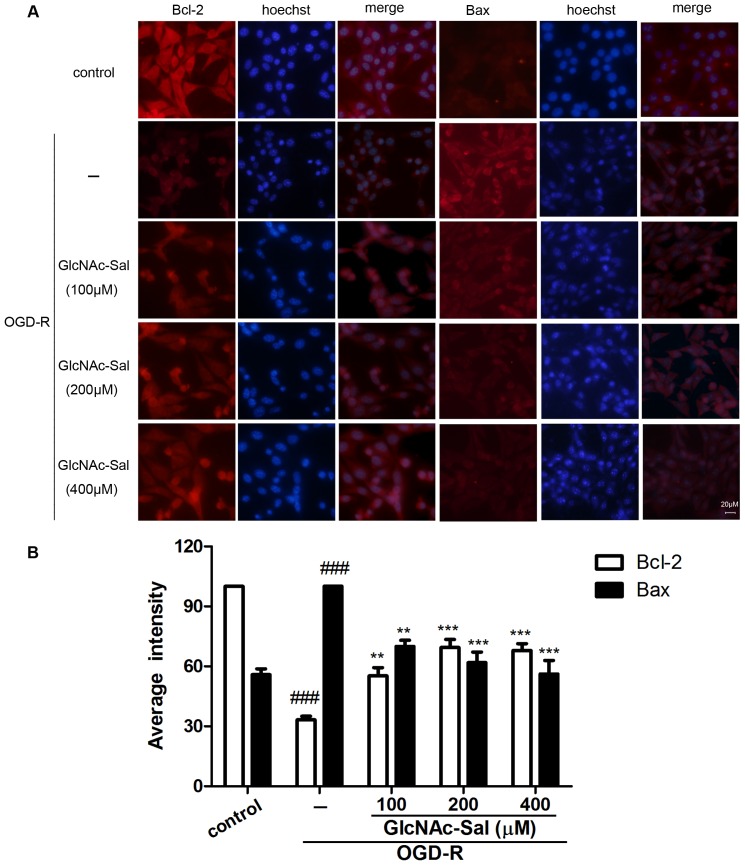
Immunofluorescence micrographs showing the expression of Bax and Bcl-2 in HT22 cells exposed to OGD-R. (A) Fluorescence staining of Bcl-2 and Bax in HT22 cells. (B) Quantification of the staining intensity of Bcl-2 and Bax. ###*p*<0.001 versus control, **p<0.01, ***p<0.001 versus OGD-R group. All data were expressed as mean ± SEM of three independent experiments.

HT22 cells were treated with GlcNAc-Sal for 24 h before exposure to OGD-R and the expression of Bcl-2 and Bax was evaluated by immunofluorescence analysis. OGD-R increased Bax protein levels and downregulated Bcl-2 expression in HT22 cells (Fig. 4A and 4B) compared with the control group. Pretreatment with GlcNAc-Sal reversed the effect of OGD-R, significantly increasing Bcl-2 expression and decreasing Bax expression.

**Figure 5:**
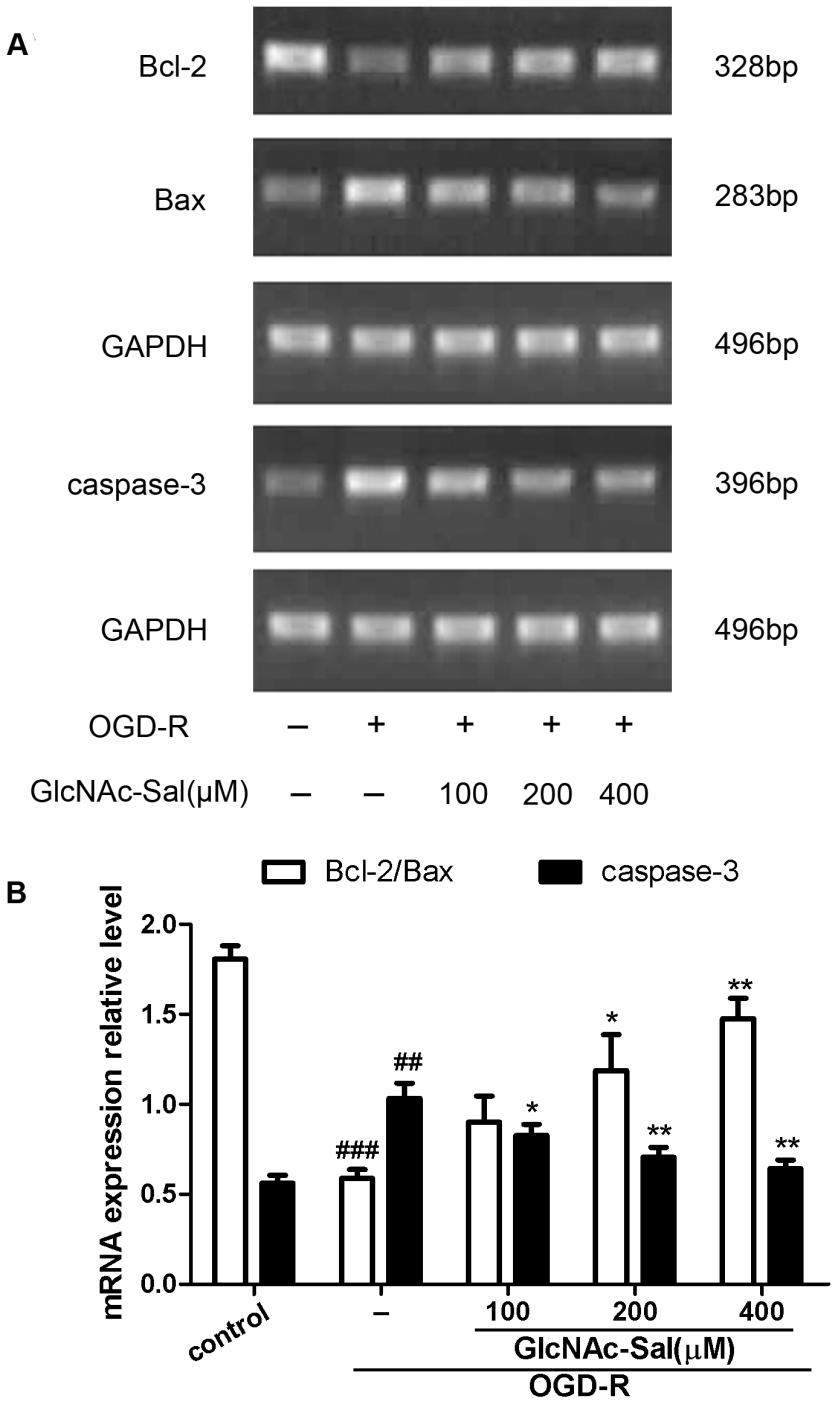
GlcNAc-Sal reverses the effect of OGD-R on the mRNA expression of caspase-3, Bcl-2, and Bax in HT22 cells. (A) Gel images showing the mRNA levels of caspase-3, Bcl-2, and Bax analyzed by RT-PCR. (B) Relative mRNA levels were quantified by normalizing to the level of glyceraldehyde-3-phosphate dehydrogenase (GADPH). Data are expressed as mean ± SEM from three independent experiments. ##*p*<0.01, ###*p*<0.001, compared with control; *p<0.05, **p<0.01, compared with OGD-R.

Reverse transcription-PCR analysis was used to examine the changes in the mRNA levels of Bcl-2, Bax and caspase-3 (Fig. 5A and B). Exposure to OGD-R alone reduced Bcl-2/Bax mRNA levels from 1.81±0.07 to 0.59±0.05 and increased caspase-3 mRNA levels from 0.56±0.04 to 1.03±0.08. GlcNAc-Sal (200 and 400 µM) pretreatment significantly inhibited the reduction of the Bcl-2/Bax mRNA ratio induced by OGD-R (1.19±0.20 and 1.48±0.11 versus 0.59±0.05). GlcNAc-Sal (100, 200 and 400 µM) also inhibited the OGD-R-induced up-regulation of caspase-3 mRNA (0.83±0.06, 0.71±0.06, and 0.64±0.05 versus 1.03±0.08).

**Figure 6:**
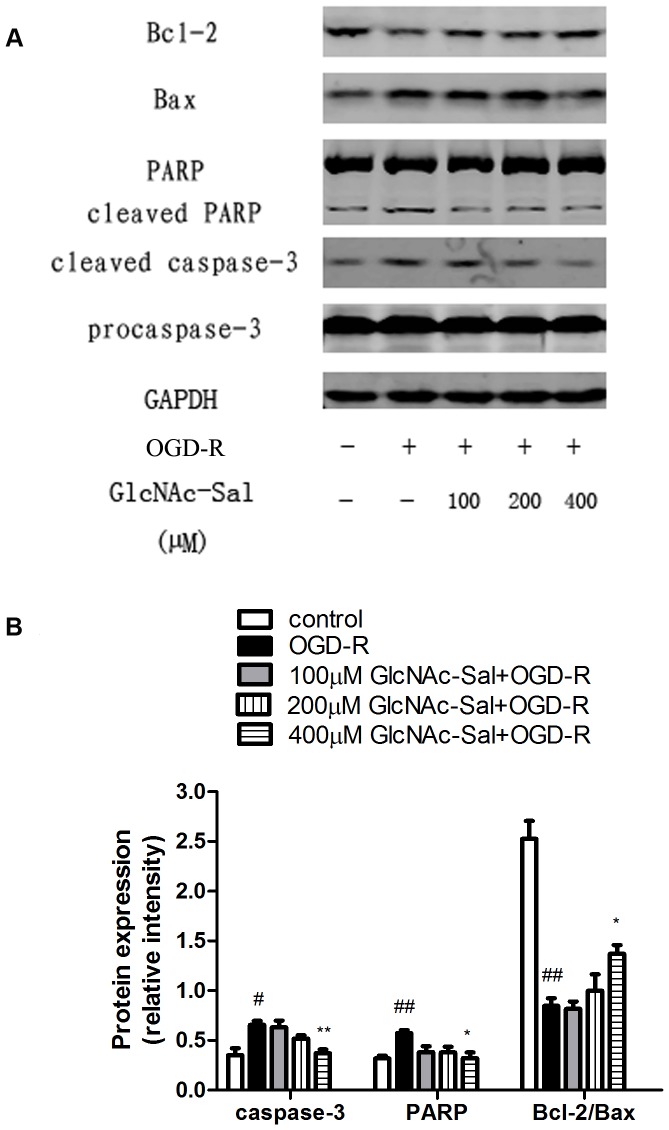
Effects of GlcNAc-Sal on the protein levels of Bcl-2, Bax, caspase-3 and PARP in OGD-R-treated HT22 cells. (A) The expression levels of Bcl-2, Bax, caspase-3 and PARP were analyzed by Western blotting. Anti-GAPDH antibody was used for normalization. (B) The intensity of bands was quantified by densitometric analysis. All values represent mean ±SEM of three independent experiments. ^#^ *p*<0.05, ^##^ *p*<0.01 vs. control; *p<0.05, **p<0.01 vs. OGD-R alone.

To further examine the signaling pathways involved in the neuroprotective effect of GlcNAc-Sal against the OGD-R insult in HT22 cells, the expressions of Bcl-2, Bax, caspase-3 and PARP were assessed by Western blotting. OGD-R treatment reduced the Bcl-2/Bax ratio and upregulated the expression of caspase-3 and PARP (Fig. 6A and B), whereas pretreatment with GlcNAc-Sal significantly increased the Bcl-2/Bax ratio and decreased the expression of caspase-3 and PARP.

### GlcNAc-Sal pretreatment attenuates OGD-R-induced ROS production in HT22 cells

**Figure 7:**
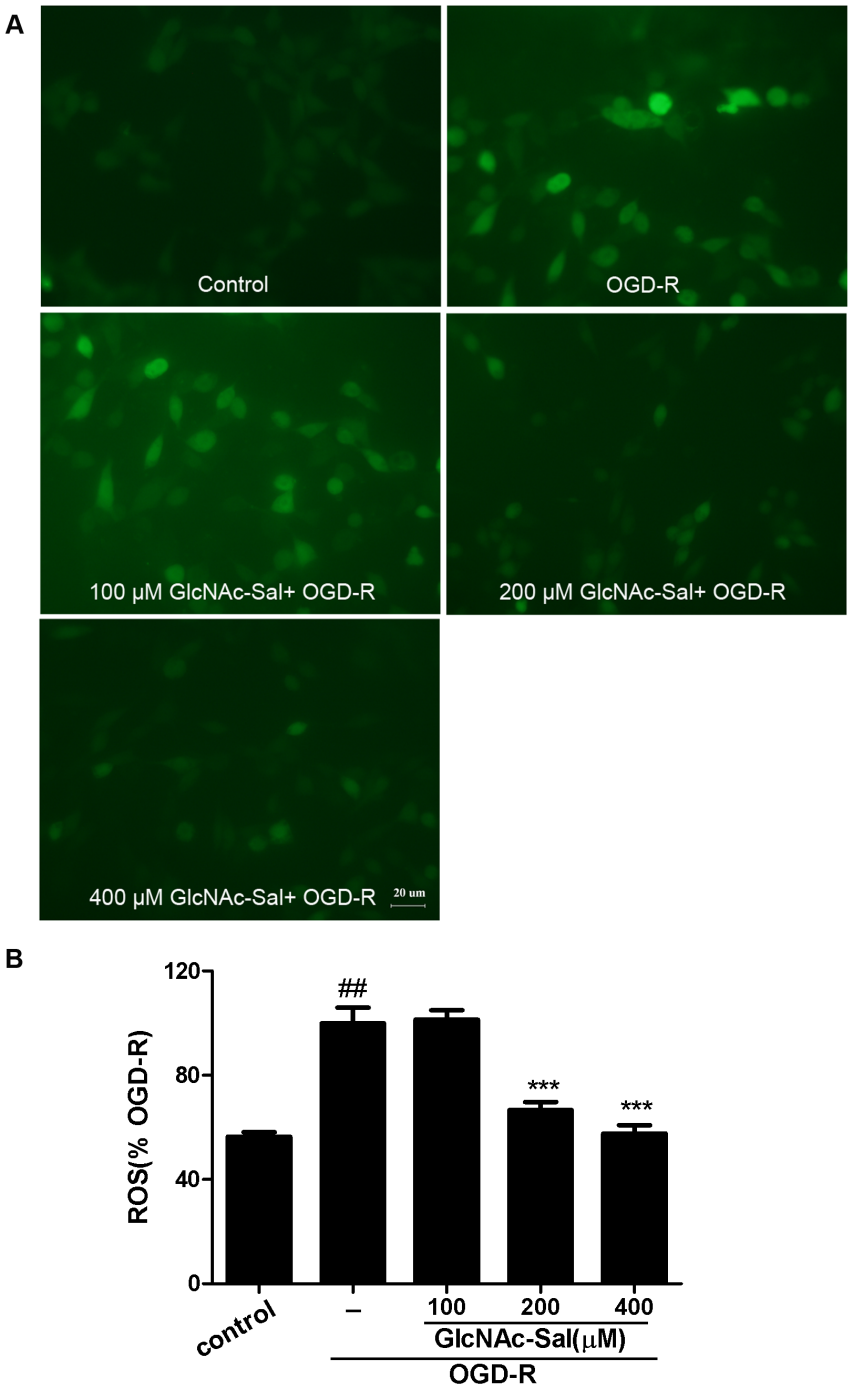
GlcNAc-Sal inhibits OGD-R-induced intracellular ROS accumulation. (A) DCF-fluorescence induced by OGD-R in the presence and absence of different concentrations of GlcNAc-Sal. (B) Quantitative analysis of DCF fluorescence intensity. Results are expressed as percent of the OGD-R-treated group. Data are expressed as mean ±SEM from three independent experiments. ##*p*<0.01, compared with control; ***p<0.001, compared with OGD-R.

Intracellular ROS production was detected by DCFH-DA. As shown in Fig. 7A and B, ROS levels were significantly increased by 177.15% by OGD-R stimulation. GlcNAc-Sal pretreatment (200, and 400 µM) dose-dependently reduced ROS generation to 66.67% and 57.70% of the OGD-R-treated group, while 100 µM GlcNAc-Sal had no effect on the intracellular concentration of ROS.

### GlcNAc-Sal inhibits OGD-R-induced NO production, NO-induced damage and the expression of iNOS in HT22 cells

**Figure 8:**
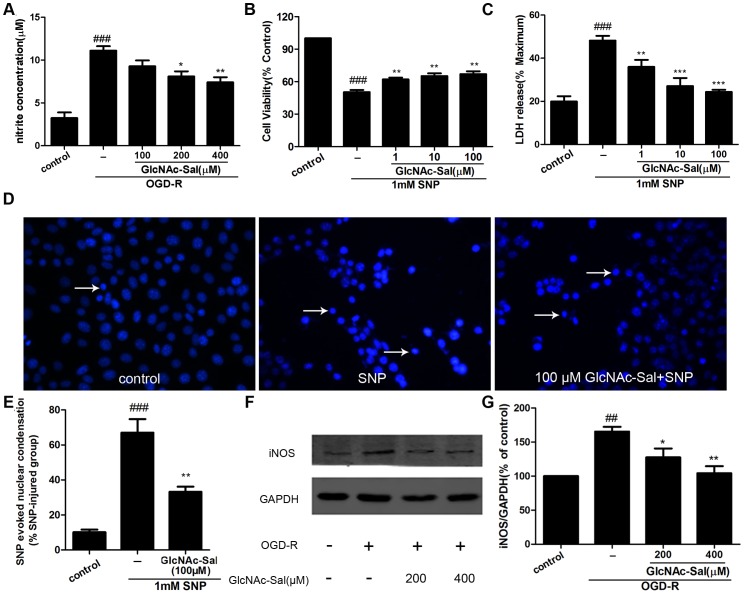
GlcNAc-Sal inhibits NO production, NO-induced damage and the expression of iNOS in OGD-R-treated HT22 cells. (A) The Griess reagent was used to evaluate the content of NO in the culture supernatant after OGD-R treatment in the presence and absence of GlcNAc-Sal. Quantitative assessment was performed using NaNO_2_ as a standard. The effect of GlcNAc-Sal on the cell viability of HT22 cells exposed to SNP stimulation was analyzed by the MTT assay (B) and LDH release assay (C). (D) The morphological features of apoptosis in HT22 cells were detected by Hoechst 33342 staining. Arrowheads indicted apoptosis cells. (E) The rates of nuclear condensation were calculated in comparison to treatment with SNP alone. (F) Effect of GlcNAc-Sal on the expression of the iNOS protein induced by OGD-R treatment. GAPDH was used for normalization. (G) The intensity of bands was quantified by densitometric analysis. The data are expressed as mean ± SEM from three independent experiments. ##*p*<0.01, ###*p*<0.001 vs. control group; *p<0.05, **p<0.01, ***p<0.001 vs. OGD-R group or SNP group.

NO production during OGD-R-induced cell death and the protective effect of GlcNAc-Sal were evaluated using the Griess reagent. As shown in Fig. 8A, OGD-R stimulation increased NO production to 343.56% of the control. Treatment with GlcNAc-Sal at 100, 200 and 400 µM concentration-dependently reduced OGD-R-induced NO production to 83.56%, 72.88% and 66.64%, respectively, compared with the OGD-R-treated group. To investigate the effect of GlcNAc-Sal on SNP-induced cytotoxicity, HT22 cells were pretreated with 1, 10, or 100 µM GlcNAc-Sal for 24 h followed by the addition of 1 mM SNP and incubation for an additional 12 h, which restored cell viability to 62.09±1.62%, 65.13±2.52%, and 67.00±2.60%, respectively, from 50.37±1.94% (Fig. 8B). The cytoprotective effect of GlcNAc-Sal was verified by the LDH release assay (Fig. 8C), which showed that GlcNAc-Sal (1, 10, or 100 µM) pretreatment reduced SNP-mediated LDH release from 48.19±2.20% to 35.99±3.23%, 27.07±3.74%, and 24.40±0.98%, respectively. The nuclear Hoechst 33342 staining assay showed that pretreatment with GlcNAc-Sal reduced the rate of nuclear condensation induced by SNP treatment (Fig. 8D and E). Western blot analysis showed that OGD-R upregulated iNOS expression compared with untreated controls (Fig. 8F and 8G) and this increase was blocked by GlcNAc-Sal pretreatment.

### GlcNAc-Sal pretreatment attenuates neuronal death and expression of apoptosis-related proteins after ischemia-reperfusion in the hippocampal region

**Figure 9:**
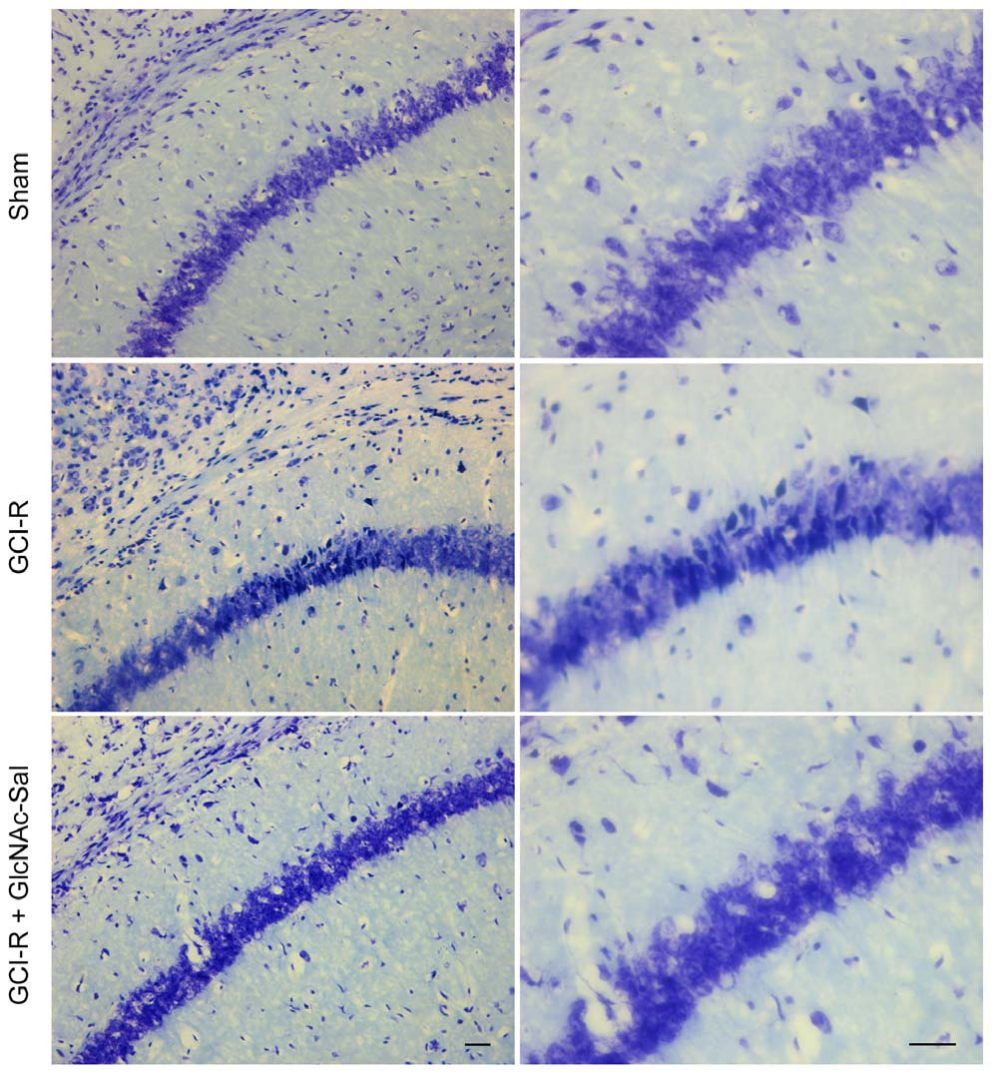
Effect of GlcNAc-Sal on GCI-R induced morphologic changes in the hippocampus of mice. Representative microphotographs of Nissl staining in the hippocampal region. Right columns show the magnification of the rectangular region in the left columns. Bar = 20 µm.

Nissl staining showed significant neuronal damage in the hippocampal CA1 region (Fig. 9). In the GCI-R group, most cells in ischemic CA1 were reduced in size with increased intercellular space indicative of injury induced by ischemia. While neuronal damage in the GlcNAc-Sal group was less severe than in the GCI-R group.

**Figure 10:**
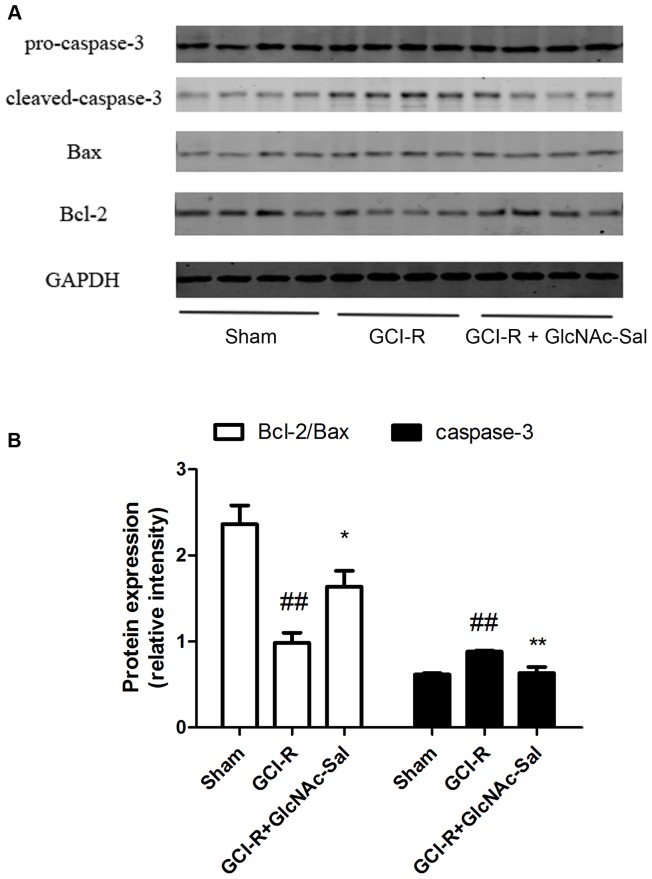
Effects of GlcNAc-Sal on the protein levels of Bcl-2, Bax and caspase-3 after GCI-R. (A) Representative Western blots show the levels of Bcl-2, Bax and caspase-3 in the hippocampus after GCI-R in the presence or absence of GlcNAc-Sal. Anti-GAPDH antibody was used for normalization. (B) Band intensity was quantified by densitometric analysis. All values represent mean ± SEM of three independent experiments. ^##^ *p*<0.01 vs. sham; *p<0.05, **p<0.01 vs. GCI alone.

Histochemical studies showed that GlcNAc-Sal had protective effects against cerebral ischemia-reperfusion insult, then we further examined the possible involvement of Bcl-2, Bax and caspase-3 in GlcNAc-Sal–mediated neuroprotection. As shown in Fig. 10, the level of caspase-3 increased significantly (0.81±0.03 to 1.04±0.09) and Bcl-2/Bax ratio decreased significantly (2.36±0.22 to 0.98±0.12) in response to GCI-R. However, pretreatment with GlcNAc-Sal decreased the level of caspase-3 (1.04±0.09 to 0.62±0.03) and increased the level of Bcl-2/Bax (0.98±0.12 to 1.64±0.18).

## Discussion

Previous study showed that, GlcNAc-Sal pretreatment significantly inhibited cell apoptosis, here we further demonstrate the neuroprotective properties of GlcNAc-Sal pretreatment in the cultured hippocampal cells as well as in mice subjected to ischemia-reperfusion injury [24], [25], [26]. Immortalized neuronal HT22 cells is a subclone of HT4, originating from mouse hippocampus, that has become a widely used in vitro model in recent years for studying the behavior of hippocampal neurons [33], [34], [35]. First we investigated the neuroprotective properties of GlcNAc-Sal in cultured HT22 cells. Pretreatment with GlcNAc-Sal, which had no significant effect on cell viability under normal conditions, reversed the injury induced by OGD-R in a concentration-dependent manner. Then, we examined the protective effects of GlcNAc-Sal in vivo using a model of transient global cerebral ischemia in mice induced by a 1 h BCCAO and 4 h reperfusion, and histochemical studies revealed that GlcNAc-Sal inhibited the neuronal damage induced by GCI-R. Taken together, our findings clearly indicated that GlcNAc-Sal pretreatment has neuroprotective effects, which prompted further investigation of the underlying mechanisms. Whether treatment with GlcNAc-Sal during or after the induction of OGD-R or GCI-R has protective effects remains uncertain and requires further study.

OGD-R model, a highly reproducible and appropriate in vitro model of ischemic stroke, is believed to better mimic the pathological conditions of stroke including excitotoxicity, oxidative stress, intracellular calcium overload, inflammation and apoptosis [36], [37], [38], [39]. In this study, morphological examinations indicated that exposure to OGD-R led to extensive apoptotic-like cell death in HT22 cells. These results are consistent with the previously reported findings that stimulation with OGD-R induces neuronal death in a prevailing form of apoptosis under in vitro conditions [40], [41].

Apoptosis, which plays a significant role in the pathophysiology of cerebral ischemia reperfusion injury [42], [43], occurs via a cascade of cellular events involving several apoptosis-regulatory genes, which are induced in apoptotic cells. The Bcl-2 family proteins represent a critical checkpoint in major apoptotic signal transduction cascades, acting upstream of irreversible damage to cellular constituents [44], [45]. The Bcl-2/Bax ratio is a determining factor in the regulation of apoptotic cell death [46]. Caspase-3, one of the key executioners of apoptosis [47], is essential for DNA fragmentation and the morphological changes associated with apoptosis [48]. Caspase-3 is activated during apoptosis and is responsible for the processing of cellular proteins including the cleavage of poly (ADP ribose) polymerase (PARP) to generate 85 and 31 kDa fragments [49]. PARP cleavage is therefore a hallmark of apoptosis [50]. In the present study, OGD-R treatment resulted in decreased expression of Bcl-2/Bax ratio at both mRNA and protein levels, activation of caspase-3 and cleavage of PARP in HT22 cells. Pretreatment with GlcNAc-Sal restored the Bcl-2/Bax ratio, inhibited caspase-3 activation and PARP cleavage. Furthermore, GCI-R also resulted in a decrease in the Bcl-2/Bax ratio and activation of caspase-3, which were reversed by GlcNAc-Sal pretreatment. Taken together, our results suggest that the inhibition of cell apoptosis may be one of the mechanisms for the neuroprotective effect of GlcNAc-Sal.

Another important mechanism leading to neuronal death during brain ischemia reperfusion is the overproduction of ROS, which may induce cell damage either directly by interacting with and destroying cellular proteins, lipids and DNA, or indirectly by affecting normal cellular signaling pathways and gene regulation [51], [52], [53]. The effect of antioxidants and ROS scavengers on reducing tissue damage following ischemic injury has been demonstrated previously [54], [55]. In the present study, pretreatment with GlcNAc-Sal effectively blocked OGD-R induced ROS production, indicating that the neuroprotective effects of GlcNAc-Sal may be mediated by inhibition of intracellular ROS generation.

Nitric oxide, a gaseous free radical and ubiquitous neurotransmitter, is one of the contributor to ROS generation. Studies have shown that NO concentrations in the brain increase from a baseline of less than 1 nM to 20 nM and even to 1–4 mM in response to cerebral ischemia/reperfusion in adult rats [56], and this increase contributes to brain damage [57], [58]. The inhibition of NO generation can reduce the size of brain infarct caused by middle cerebral artery occlusion (MCAO) in rats [59]. Our results showed that OGD-R induced the overproduction of NO, which was partially restored by GlcNAc-Sal treatment. To further investigate the effect of GlcNAc-Sal on the induction of apoptosis by NO overproduction, we used SNP to build a neurotoxic model of HT22 cells. The results revealed that GlcNAc-Sal significantly prevented SNP-induced injury. NO is generated by iNOS (inducible NO synthase), which is expressed in *in vitro* [60] and *in vivo* models of brain ischemia [61]. Evidence has shown that iNOS inhibitors and antioxidants prevent ischemia-induced neurotoxicity through NO-mediated mechanisms [1], [10], [62], [63]. Analysis of the expression of iNOS showed that GlcNAc-Sal significantly prevented the upregulation of iNOS caused by OGD-R stimulation. These data suggest that the protective mechanisms of GlcNAc-Sal in OGD-R-induced damage in HT22 cells are related to the inhibition of iNOS-derived NO production and NO-related apoptotic cell death.

Scholars found that ROS appear to play a particularly important role in triggering changes that lead to neuronal apoptosis during brain ischemia. And NO can react with O_2_^−^ to produce peroxynitrite, which is a strong oxidative radical that probably involved in neuronal apoptosis following cerebral ischemia [38]. Therefore, in this work we speculated that the neuroprotective mechanism of GlcNAc-Sal may directly inhibit apoptosis-related protein or indirectly act on inhibiting ROS and NO generation involved in the inhibition of apoptosis.

## Conclusions

This study demonstrates that GlcNAc-Sal pretreatment prevents OGD-R-induced cell damage in cultured HT22 cells in vitro and GCI-R induced injury in vivo by the direct or indirect suppression of cell apoptosis. These findings indicate that GlcNAc-Sal may reduce the spread of ischemia injury and rescue the dying cells in the ischemia region. It could be developed as a neuroprotectant to brain ischemia.
